# Supplementary material for: Identification and Characterization of Human Monoclonal Antibodies for Immunoprophylaxis against Enterotoxigenic Escherichia coli Infection
Source: Infect Immun. 2018 Jul 23;86(8):e00355-18. doi: 10.1128/IAI.00355-18 (PMC6056861; doi:10.1128/IAI.00355-18)
Supplement: Supplemental material [file supp_86_8_e00355-18__index.html]

Supplemental material 

# Identification and Characterization of Human Monoclonal Antibodies for Immunoprophylaxis against Enterotoxigenic Escherichia coli Infection

## Supplemental material

- Supplemental file 1 -

  Legend for Fig. S1.

  PDF, 7.3K
- Supplemental file 2 -

  Fig. S1. Characterization of dimeric and secretory IgA.

  PDF, 30K
