# Supplementary material for: Identification and Characterization of Human Monoclonal Antibodies for Immunoprophylaxis against Enterotoxigenic Escherichia coli Infection
Source: Infect Immun. 2018 Jul 23;86(8):e00355-18. doi: 10.1128/IAI.00355-18 (PMC6056861; doi:10.1128/IAI.00355-18)
Supplement: Supplemental material [file IAI.00355-18_zii999092505s1.pdf]

Figure S1. Characterization of dimeric and secretory IgA. Panel A, Size exclusion chromatography profiles of purified 68-61 sIgA2 (blue) and 68-61 dIgA2 (orange). The single peak of each sample demonstrates >97% purity. SDS-PAGE of sIgA, dIgA, and IgG of 68-61 HuMAb. All samples were run on a NuPage 3-8% Tris-Acetate gel and stained with SPYRO Ruby.
