# Supplementary figures and images for: Identification and Characterization of Human Monoclonal Antibodies for Immunoprophylaxis against Enterotoxigenic Escherichia coli Infection
Source: Infect Immun. 2018 Jul 23;86(8):e00355-18. doi: 10.1128/IAI.00355-18 (PMC6056861; doi:10.1128/IAI.00355-18)

**A**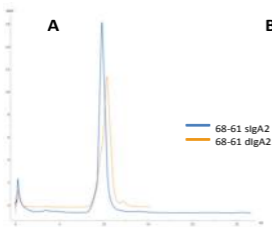**B**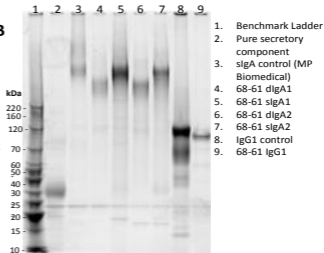

Supplement: Supplemental material [file IAI.00355-18_zii999092505s2.pdf]
